# Adapting Imaging Protocols for PET-CT and PET-MRI for Immunotherapy Monitoring

**DOI:** 10.3390/cancers13236019

**Published:** 2021-11-30

**Authors:** Bettina Beuthien-Baumann, Christos Sachpekidis, Regula Gnirs, Oliver Sedlaczek

**Affiliations:** 1 Radiologie, Deutsches Krebsforschungszentrum Heidelberg, Im Neuenheimer Feld 280, 69120 Heidelberg, Germany; r.gnirs@dkfz-heidelberg.de (R.G.); o.sedlaczek@dkfz-heidelberg.de (O.S.); 2 Klinische Kooperationseinheit Nuklearmedizin, Deutsches Krebsforschungszentrum, Im Neuenheimer Feld 280, 69120 Heidelberg, Germany; c.sachpekidis@dkfz-heidelberg.de; 3 Klinik für Diagnostische und Interventionelle Radiologie, Universitätsklinikum Heidelberg, Im Neuenheimer Feld 420, 69120 Heidelberg, Germany

**Keywords:** PET-CT, PET-MRI, immunotherapy

## Abstract

**Simple Summary:**

In this review, we discuss the possible advantages and methods to overcome potential obstacles of applying combined imaging protocols of PET-CT and PET-MRI, within the context of staging and restaging of patients under immunotherapy, in order to achieve “multi-hybrid imaging” in one single visit.

**Abstract:**

Hybrid imaging with positron emission tomography (PET) in combination with computer tomography (CT) is a well-established diagnostic tool in oncological staging and restaging. The combination of PET with magnetic resonance imaging (MRI) as a clinical scanner was introduced approximately 10 years ago. Although MRI provides superb soft tissue contrast and functional information without the radiation exposure of CT, PET-MRI is not as widely introduced in oncologic imaging as PET-CT. One reason for this hesitancy lies in the relatively long acquisition times for a PET-MRI scan, if the full diagnostic potential of MRI is exploited. In this review, we discuss the possible advantages of combined imaging protocols of PET-CT and PET-MRI, within the context of staging and restaging of patients under immunotherapy, in order to achieve “multi-hybrid imaging” in one single patient visit.

## 1. Technical Background Hybrid PET

Following the development of the first commercial hybrid scanner in 2001, positron emission tomography-computed tomography (PET-CT) [1] was readily implemented into the clinical routine for staging and therapy response assessment in the oncological setting. The combination of the functional information provided by PET with the morphologic detail of CT, acquired in an identical patient position, promoted the wide acceptance of PET-CT in clinical routine. PET-CT imaging involves CT acquisition—low dose CT for attenuation correction or diagnostic CT including contrast media—which is performed within few breath holds. The PET acquisition is performed with advanced PET-scanner technology (i.e., time-of-flight, continuous bed motion, silicon-photomultiplier) [2], and after application of ^18^F-fluorodeoxyglucose (^18^F-FDG)—still the workhorse of PET imaging—or a variety of radiotracers reflecting different molecular or pathophysiologic parameters. A main strength of PET-CT is the performance of whole-body imaging in a reasonable time-frame, with the total acquisition time of a scan (from the base of the skull to mid-thigh), nowadays, not exceeding 30 min.

**Figure 1:**
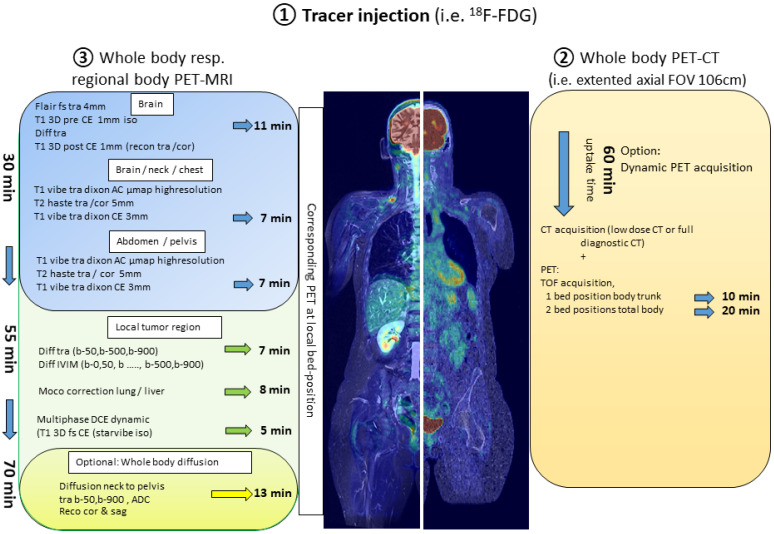
Components of a “multi-hybrid-imaging” oncological protocol combining PET-CT and PET-MRI with one single application of the radiopharmaceutical. The examination protocol would begin with an initial whole-body PET-CT lasting between 10 and 20 min (right side of the figure) followed by PET-MRI. PET-MRI can be performed with a fast whole-body acquisition completed within less than 30 min (upper part of the image depicting the PET-MRI protocol—blue background). To significantly take advantage of the MR-component, local PET-MRI imaging of defined body regions lasting approximately 20 min (middle part of the image depicting the PET-MRI protocol—green background) can be added. Finally, the PET-MRI examination can be further expanded with the generation of DWI of the body trunk with a minimum acquisition time of 13 min (lower part of the image depicting the PET-MRI protocol—yellow background).

Presently, the PET-CT technology further advances the introduction of “total-body” technology, which consists of a vast extended axial field of view by combining multiple PET-rings covering the head and the body trunk or even the head to toe [3,4]. Due to this large axial field of view, the detection of annihilation events and the resulting sensitivity is maximized. This opens up an opportunity to substantially shorten the imaging protocol (less than 10 min for the entire axial field) or to considerably reduce the applied activity with still short-ranged acquisition times of modern PET-CT technology [5]. Furthermore, the dynamic acquisition with an extended body coverage can be finally achieved, facilitating dosimetry assessments when exploring novel PET tracers, and potentially reintroducing a broader interest in absolute quantification of tumor and organ/tissue metabolism as part of therapy response assessment (Figure 1).

Approximately 10 years ago, simultaneous hybrid PET-magnetic resonance imaging (PET-MRI) was introduced for clinical use. Replacing CT with MRI carries some important advantages. In contrast to CT, MRI offers superb soft tissue contrast and additional functional tissue characterization by means of the multiple available MR-sequences, such as diffusion weighed (DWI) and dynamic contrast enhanced (DCE) imaging. In this context, the merge of MRI with PET imaging offers the opportunity to extract a bundle of imaging biomarkers from one single examination protocol. Importantly, unlike CT, information from MRI comes without further ionizing radiation, which is particularly relevant in the pediatric population and in women of child-bearing age [6]. Although the prospective knowledge gain through the combination of PET and MRI is still thrilling, serious technical hurdles had to be solved before the first commercial hybrid PET-MRI was introduced. Both, the interaction of the magnetic field on the photomultiplier and on the PET detectors had to be addressed. Initially, concepts comprised sequential imaging by the combination of PET-CT with an MRI in the next room, while the patient was moved between the scanner on a mobile imaging couch [7] or positioning the PET ring and the MRI at the opposite end of a rotating imaging table [8]. True simultaneous acquisition of PET and MRI was finally accomplished by integrating the PET ring into the MRI between the MRI body coil and the gradient coils. This became possible by replacing the classic photomultiplier by avalanche photodiodes, which are less susceptible to the magnetic field [9].

With PET-CT, the attenuation correction of the patient body for PET emission can be derived from the CT data. However, in PET-MRI, the attenuation correction of the PET data is a strenuous technical issue, which is continuously a subject of improvement. For PET-MRI, the attenuation map has to be constructed from the MRI. T1-weighed data are segmented into distinct tissue classes (fat, soft tissue, background air, lung tissue) and respective attenuation values are assigned. Bony structures pose a problem, since bone has almost no signal in MRI. In former reconstruction algorithms, bone was not considered in the attenuation map. More recent algorithms include bone structures based on atlas-derived templates. This atlas-based attenuation reconstruction model addresses the systematic underestimation of attenuation when disregarding the attenuation by bone [10], but the accuracy of the model still has to be observed when applied on a broad scale.

The number of oncological studies investigating the role of PET-MRI, mainly in comparison to PET-CT, is continuously increasing. Most of these studies have documented the equivalence of the two hybrid imaging methods in terms of diagnostic accuracy [11,12]. However, in clinical routine, PET-MRI is not as widely adopted as PET-CT since it also carries some significant disadvantages. In addition to the cost of the scanner, one hindrance are the long acquisition protocols of PET-MRI. In the following, only the duration of the scan time on PET-MRI is considered, since the preparation time (tracer injection and time for tracer distribution before the start of the scan) of, i.e., an oncological ^18^F-FDG-PET application for staging or restaging purposes, is similar for PET-CT and PET-MRI. In contrast to PET-CT, where the PET acquisition covers the major part of the scan duration, in PET-MRI, the MRI part is likely to be the time-dominant modality. This is justified by the fact that in order to fully exploit the advantages of diagnostic MRI, a minimum set of sequences, addressing specific tissue characteristics, are acquired at every bed position. Starting with the cranium, the composite of T2-weighted imaging with fat-saturation, DWI, and T1-weighed 3D dataset pre- and post-contrast already require at least 11 min of acquisition time. For four bed positions covering the body trunk, the minimal structural imaging would include a single-shot T2-weighted sequence and a T1-Vibe–Dixon (LAVA/Thrive) pre- and post- contrast with another 6–8 min per position. Moreover, in the oncological setting, the generation of DWI of the body trunk is almost compulsory, which leads, however, to further extension of the acquisition time (minimum of 12–13 min). Therefore, a RECIST conform whole body scan (i.e., contrast-enhanced axial MRI including T1 and T2 slices with a slice thickness of ≤5 mm) rarely lasts less than 60 min. Notably, this estimation is made without taking into account potential, additional dynamic acquisitions after the application of contrast agent (simple three phase acquisitions or DCE) for imaging of specific tissues or body areas, such as the liver, primary tumor, etc. (Figure 1). Already in the advent of PET-MRI, the optimization of scan protocols in accordance to specific patient groups (i.e., children) and disease cohorts (i.e., different tumor entities) has been discussed [13,14,15,16]. Evidently, considering—the still rather seldom performed—scans with PET radiopharmaceuticals labeled with tracers of longer half-lives, such as Zirconium-89 (^89^Zr; half-life 3.3 days), well matched to the circulation half-lives of antibodies and thus, potentially suitable for immunotherapy monitoring, the scan duration of PET might exceed the time necessary for the MRI sequences [17,18].

With regard to shortening the MR scanning time, the implementation of MRI fingerprinting could be a major step. This technique provides an accelerated acquisition of data, which delivers multiple property maps in the time it would take to acquire only one map when using conventional methods [19]. Furthermore, the quantitative approach of fingerprinting in addition to the traditionally qualitative information read from the different MRI sequences would be a favorable addition to functional PET. Currently, fingerprinting in patient studies has been performed in limited body regions and/or disease entities (i.e., brain, cardiac, breast, prostate). This could be particularly useful when PET-MRI scanning is performed over specific body regions after completion of whole body PET-CT. At the same time, a fast whole-body MRI in the fingerprinting technique will be challenging due to the generation of large data volume, the necessity to construct a whole-body dictionary as a reference dataset, long reconstruction times, and the extensive motion correction. In this context, except for the brain or very distinct regions, the implementation of fingerprinting into the workflow of PET-MRI cannot be expected in the close future for routine clinical scans, and will have to be further developed and investigated in the research setting.

However, several acceleration techniques are already in use, some of which can be expected to show up in PET-MRI scanners soon, including parallel acquisition techniques, golden-angle radial sparse parallel technique, simultaneous multi-slice acceleration, and even artificial intelligence (AI)-powered image reconstruction technologies [20].

## 2. Combining Protocols of PET-CT and PET-MRI

Regarding PET-MRI, the question that inevitably arises is, if there is an added benefit in the management of oncological patients, especially in diagnostic centers or departments, in which PET-CT is already available. The answer is most probably not a simple one. Although, a combination of whole-body hybrid imaging with both PET modalities would result in a plethora of potentially beneficial, complementary, morphologic, metabolic, and functional information, issues related to patient tolerance (acceptance), as well as logistical considerations with regard to the workflow of the department may render this attempt relatively impractical at first sight. This problem may be solved with the application of modified, faster PET-MRI acquisition protocols. These workflows include, for example, an initial whole-body PET-CT (optimally with new generation scanners offering faster whole-body coverage) followed by fast whole-body PET-MRI acquisitions, which can be completed within less than 20 and 30 min, respectively, within one single radiotracer injection [15,16]. Another algorithm involves dedicated local PET-MRI imaging of defined body regions (typically the main tumor region and the brain, only). Considering that MRI is the anatomic imaging modality of choice in certain cancers, it would be reasonable to combine whole-body PET-CT with regional PET-MRI including full diagnostic and functional MRI-coverage of the distinct body/tumor region. Advantages of this combination of hybrid PET modalities are evident, involving the superiority of CT, e.g., for lung parenchyma imaging—regarding both the detection of small noduli and other pathological conditions, such as treatment-related side effects—and, at the same time, the advantages of MRI in the morphologic evaluation of organs, such as the bone, liver, and brain parenchyma. Combining the workflows of both hybrid scanners would require a high level of strategic planning, but these protocols will very likely optimize the diagnostic gain. For the patient, this combination is feasible with no additional radiation burden and minimal extra time, since it would be performed with only one injection of radioactivity and require only one visit of the diagnostic department (Figure 1).

**Figure 2:**
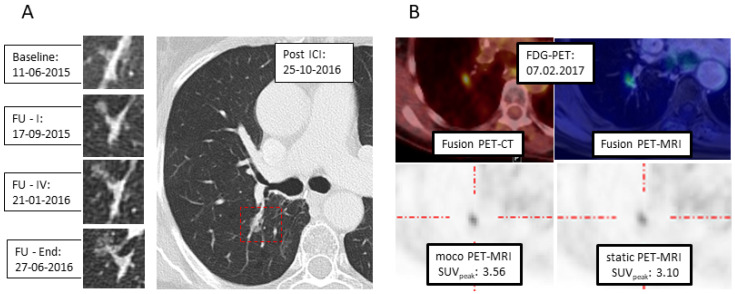
Example of respiratory motion correction in PET-MRI. (**A**) CT: Development of a semi-solid formation post-immunotherapy at the right hilum. (**B**) ^18^F-FDG-PET with PET/CT (upper left) und PET-MRI (upper right): With MRI-based motion correction (moco), a higher SUV_peak_ is calculated (lower left), compared to static reconstruction (lower right).

Motion correction is, moreover, one of the fields where this combined scanning could play a role: Although PET acquisitions are conducted with shallow breathing, still, organs and especially tumors in the lung are moving during the scan, which might induce blurring of the region of interest and inaccuracy in quantification. It has long been the aim to correct PET data for motion. With PET-MRI, motion correction has been introduced by monitoring, i.e., the lung motion by sagittal star-vibe sequences, acquired simultaneously to the PET acquisition [21]. One example is displayed in Figure 2. In this patient, the semi-solid nodular structure in the right lung, which developed slowly after immunotherapy, was further characterized as suspicious tumor recurrence/secondary cancer by ^18^F-FDG-PET-CT. Immediately after PET-CT, the patient was transferred to PET-MRI to establish a motion corrected baseline SUV-quantification for eventual further treatment regimen evaluation.

## 3. Hybrid PET and Immunotherapy

In recent years, a shift in systemic oncologic treatments towards immunotherapy has taken place. In particular, the clinical introduction of immune checkpoint inhibitors (ICIs) has altered the landscape in the management of different tumor entities, such as melanoma and lung cancer, leading to unprecedented response and survival rates [22,23]. At the same time, the advent of these novel immunotherapeutic agents has been associated with some challenges regarding the reliable assessment of response.

Driven by their unique mechanism of action, ICIs generate inflammations rather than direct tumor lysis (as conventional cytotoxic approaches), which, in turn, may lead to atypical response patterns. These patterns include, among others, the phenomena of (1) pseudo-progression, defined as an initial increase in tumor burden followed by tumor regression, (2) hyper-progressive disease, an aggressive pattern of rapid, marked disease progression associated with very poor survival, and (3) dissociated responses, characterized by the regression of some lesions and the concurrent growth of other lesions or the appearance of new ones [24,25]. These issues raise the question of how to evaluate the response to ICIs reliably and at the earliest time point during the treatment, since this would have significant therapeutic and prognostic implications for the patient, as well as socioeconomic benefits, considering that the treatment expenditure exceeds EUR 100,000/per patient [26].

**Figure 3:**
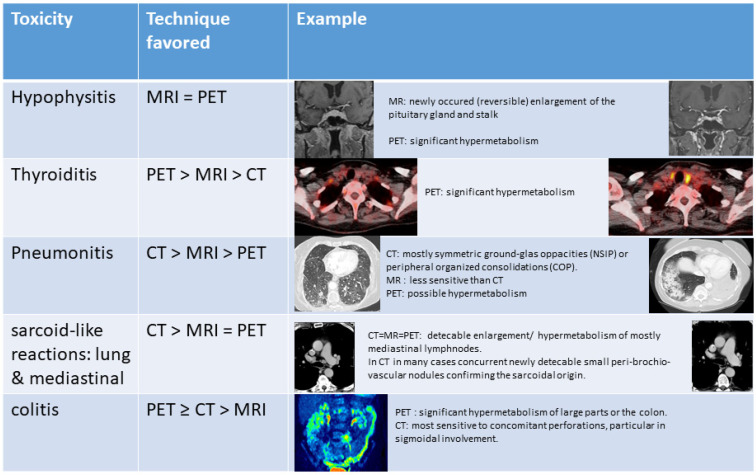
Preferred imaging modality for the characterization of immune-related side effects (PET refers to ^18^F-FDG-PET).

Moreover, ICIs are linked with the emergence of a new class of side effects, the immune-related adverse events (irAEs), which resemble autoimmune responses and may further complicate imaging assessment [27]. Concerning irAEs, the early detection of hypophysitis, thyroiditis, pneumonitis, and colitis, as well as the reliable differentiation of benign signs of immune activation, such as sarcoid-like reactions of the lung and mediastinal lymph nodes, from progressive disease, is crucial for the successful treatment and management of the patients (Figure 3).

In an attempt to provide hints for a reliable immunotherapy response assessment other than RECIST, several new response criteria, both radiological [28,29,30] and PET-based [31,32,33], have been developed. Although these criteria seem to outperform the conventional ones, there still remains uncertainty regarding the reliable differentiation between true progression and pseudo-progression or dissociated responses based exclusively on morphologic imaging features or metabolic assessment with ^18^F-FDG-PET. Concerning PET, one approach to potentially overcome this problem is the development of novel PET radiotracers specifically targeting the cytotoxic CD8+ T cells [17] or the PD-1 or PD-L1 pathways [34,35] in order to assess the T-cell content and the PD-1 and/or PD-L1 expression in tumor lesions, respectively. This information could be used supplementary to the information derived from ^18^F-FDG, since the latter—due to its nonspecific nature—accumulates in both tumor lesions and ICIs-induced sites of inflammation. These approaches are highly promising, but are still at a preliminary level [36]. In this framework, the definition of the potential role of a combined PET-CT and PET-MRI protocol in immunotherapy response monitoring is particularly challenging. Apart from the above-mentioned, more general, major strengths and weaknesses of PET-MRI, one should take into consideration the specific challenges encountered with this type of treatment and the expectations from the therein applied imaging modalities. In the clinical scenario of immunotherapy, it can be argued that the generation of complementary, comprehensive imaging information may aid in the reliable interpretation of the atypical response patterns, as well as the identification of the wide range of irAEs (Figure 3) and the investigation of signs of immune activation, which could in turn prove beneficial for the ICIs treatment response evaluation. Moreover, the lack of readily available for clinical use immunotherapy-specific radiotracers, could be partly compensated by the enrichment of our diagnostic armamentarium with a “multi-hybrid-imaging” tool, offering high-quality morphologic, functional, and metabolic information in a reasonable time-frame and a single patient visit, avoiding the long acquisition times required with these experimental tracers, which, in turn, increase the costs associated with their application.

This position is further supported by recently published data on the role of multiparametric MRI (mpMRI) in the evaluation of immunotherapy responses. In particular, in a study investigating the ICIs responses of metastatic melanoma patients using mpMRI, several direct or calculated readouts were of prognostic value, namely diffusion/ADC, the derived cell-density, and DCE derivates (K^trans^, v_e_, and v_c_) already after 3 weeks of treatment [37]. Moreover, DCE- and DWI-MRI have been shown to distinguish pseudoprogression from real progression in different tumor entities and brain metastatic disease under ICIs [38,39]. Regarding DCE-MRI, reduced capillary permeability (K^trans^) and plasma volume were detected in pseudoprogression as compared to real progression, whereas with regard to ADC, serial regional intratumoral time-courses appeared to be predictive of response to ICIs-therapy. Therefore, the combination of whole body analysis of glucose metabolism with mpMRI-derived data offered by integrated ^18^F-FDG PET-MRI may significantly aid in the early prediction of treatment response to ICIs, as has been recently documented in melanoma and lung cancer patients [40,41]. Furthermore, the potential of exploiting the high resolution data of CT in combination with the high contrast information of PET and MRI—each of which bring its own advantages in the investigation of individual organs and systems—would most probably have a beneficial effect on the reliable identification and interpretation of many irAEs and signs of immune activation (Figure 3). It is the comparison with the pre-therapeutic imaging confirming the ICI-linked emergence of the toxicity: This is true for the detection of hypophysitis in MRI, but particularly for many of the interstitial lung findings, that are overseen or misinterpreted upon first detection in any modality. Therefore, an explicit notification of both, the use of ICIs and the date of treatment initiation are crucial information for diagnostic readers.

Unfortunately, a large cohort data on the application or even more, a comparison of these different novel approaches regarding their efficiency in immunotherapy monitoring are still lacking. Furthermore, all of the advanced techniques, may they be PET-, CT- or MRI-based, always rely on the repetition of exact imaging protocols starting with a baseline scan ahead of therapy initiation and continuing during therapy assessment and follow-up. Although for some imaging scenarios the combined PET-CT and PET-MRI may seem to “overproduce” diagnostic information, this might be desirable, at least for now, until the optimal scanning protocol is defined.

## 4. Conclusions

In conclusion, despite some yet partly unaddressed challenges, the steadily growing literature in the field suggests that the combination of PET-CT and PET-MRI, performed sequentially in modified, shorter protocols, as suggested above, combines the best of both “hybrid PET worlds”. Furthermore, it has the potential to become a powerful diagnostic tool particularly in immunotherapy monitoring.
